# Non-adherence to tuberculosis contact screening and its associated factors in Kuching, Sarawak: A cross-sectional study

**DOI:** 10.51866/oa.536

**Published:** 2024-05-12

**Authors:** Ak Lis Esther Sumi, Teo Ju Yeng Audrey, Anak Ebon Brownson, Ak Disen Donna

**Affiliations:** 1 MB BCH BAO, MAFP, FRACGP, Klinik Kesihatan Tanah Puteh, Jalan Kwong Lee Bank, Kuching, Sarawak, Malaysia. Email: er8598@gmail.com; 2 MBBS, MAFP, FRACGP OHD, Certificate of Clinical Wound Care, Fellowship in Addiction Medicine, Klinik Kesihatan Sri Aman, Jalan Bukit Tembak, Sri Aman, Sri Aman, Sarawak, Malaysia.; 3 MBBS, DFM, Klinik Kesihatan Jalan Masjid, Jalan Masjid, Kuching, Sarawak, Malaysia.; 4 MBBS, DFM, Klinik Kesihatan Jalan Masjid, Jalan Masjid, Kuching, Sarawak, Malaysia.

**Keywords:** Tuberculosis (TB), Contact tracing, Adherence, Knowledge

## Abstract

**Introduction:**

Tuberculosis (TB) contacts in Malaysia undergo follow-up screening to reduce their risk of active or latent TB. However, adherence to this screening is low. Limited studies have explored the factors contributing to non-adherence to follow-up screening. This study aimed to determine the non-adherence rate and reasons in a government health clinic.

**Methods:**

Participants were TB contacts due for their 2nd contact screening (including those who attended their first contact screening at Petra Jaya Health Clinic from November 2018 to March 2019), were aged at least 18 years and were able to understand English or Malay. Data were collected during the second contact screening from August 2019 to January 2020.

**Results:**

A total of 383 TB contacts were included. Of them, 56.6% (n=217) were aged 20–39 years, and the sex distribution was equal (men: 44.1%, n=169). The majority were non-household contacts (82.2%, n=315). The rate of non-adherence to follow-up screening was 19.1% (n=73). Approximately 52.1% (n=36) reported forgetting their scheduled appointment date as the primary reason for non-adherence. The influencing factors included employment and ethnicity. Only 39.1% (n=27) were aware of their risk for active TB, while 49.5% (n=189) were unsure whether TB can be cured with proper treatment.

**Conclusion:**

The findings highlight the need to improve the reminder system for TB contacts. Although direct association between knowledge and adherence could not be established, the low percentage of correct answers to most basic knowledge questions associated with TB indicates a need to improve health education for TB contacts.

## Introduction

Tuberculosis (TB) remains a disease of public health importance in Malaysia. It is one of the top 10 causes of death worldwide and the leading cause of death from a single infectious agent, *Mycobacterium tuberculosis,* which is spread when people who are infected with TB expel bacteria into the air. TB typically affects the lungs (pulmonary TB) but can also affect other sites (extrapulmonary TB). Geographically, most TB cases in 2018 were recorded in the WHO regions of South-East Asia (44%).^1^

Effective contact tracing and investigations are important for successful TB control. TB contacts include individuals who live in the same household or share the same air space with the index patient for a reasonable duration before the index patient receives TB treatment. However, the minimum physical distance or exposure duration has not been well established.^2^

In 95 studies conducted across low- and middle-income settings, the prevalence of active and latent TB was 3.1% and 51.5%, respectively, among all TB contacts screened. The incidence was the greatest in the first 5 years after exposure. In 108 studies performed across high-income settings, the prevalence was 1.4% and 28.1%, respectively.^3^

TB contact screening in Malaysia is conducted at designated major government health clinics. In Sarawak, TB contacts are seen at 8-month intervals up to 2 years after the initial visit. Adherence to TB contact follow-up screening in Sarawak is poor, but no data have been published thus far. In Malaysia, limited studies have explored the factors contributing to nonadherence to follow-up screening in primary care settings.^4,5^ Therefore, this study aimed to determine the rate and associated factors of non-adherence to TB contact follow-up screening in a government health clinic in Sarawak, East Malaysia.

## Methods

A cross-sectional design was adopted. Participants were TB contacts who were due for their 2nd contact screening at Petra Jaya Health Clinic in the city of Kuching, the capital of Sarawak, East Malaysia. This site was chosen, as it uses an electronic TB contact data registration system.

In Sarawak, there is a slight variation in the follow-up interval and duration for TB contact screening. Malaysia’s national guideline suggests screening at the 3rd, 9th and 18th months after the initial clinic visit.^2^ However, in Sarawak, the screening interval is set at 8 months after the initial clinic visit, for a total duration of 24 months.^6^ This variation is tailored to better suit Sarawak’s geographic and logistical complexities.

Chest radiography is performed at each clinic visit, with an additional Mantoux test at the initial visit. Appointments for the subsequent 8-month screenings are written in an appointment slip given to patient by the nurse or medical assistant in charge. If patients do not turn up for their subsequent scheduled visits, the staff would first attempt to contact them (before notifying to relevant Health Inspectors). This is part of the clinical care practice that aims to re-engage TB contacts into the clinical care process.

In this study, TB contacts who were due for their 2nd TB contact screening (either those who adhered to their appointment dates [turned up to the clinic on their scheduled date] or those who did not [defaulted their 2nd scheduled TB contact screening date by 2 weeks or more, only turning up to the clinic after a phone call reminder by the clinic staff]), were aged 18 years and above and were able to understand English or Malay were included. Conversely, TB contacts who did not turn up for their 2nd TB contact screening (despite 3 or more attempts of phone call reminders by the clinic staff over 1 week), were transferred out of Kuching district during data collection, were illiterate in English or Malay and were uncontactable through a phone call were excluded.

The details of eligible TB contacts were obtained from the clinic’s existing electronic records (Microsoft Access). A universal sampling method was used. Upon arrival of TB contacts for their 2nd screening appointment at the TB clinic, the responsible staff notified one of our study team members. Our team then provided TB contacts with a participant information sheet and an informed consent form, along with detailed explanations. Upon providing consent, TB contacts received a structured and pre-tested self-administered questionnaire in their preferred language (English or Malay).

For non-adherent TB contacts (defaulted their 2nd scheduled TB contact screening date by 2 weeks or more), the clinic staff contacted them through a phone call to reschedule the appointment. When they showed up for the rescheduled appointment and agreed to participate in this study, they were then given a similar questionnaire (with an additional section on the reasons for non-adherence in their preferred language - English or Malay).

**Figure 1:**
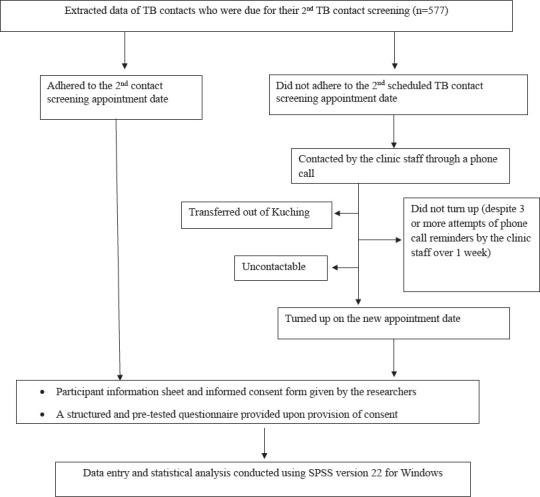
Flow chart of the study. Data collection period: August 2019 to January 2020.

The study population comprised two groups: those who adhered to their 2nd contact screening and those who did not (Figure 1).

### Sample size

The minimum sample size required was 323, calculated using the OpenEpi software based on a P-value of 0.05 and study power of 95%, with an assumed prevalence of non-adherence of 68%.^7^ The sample size was increased to 380 to cover for a 15% non-adherence rate.

### Study instrument

**Questionnaire**

Our questionnaire consisted of 3 main sections. The 1st section collected data on participants’ sociodemographic characteristics. The 2nd section, partly adapted and modified from a tool used in a study in Vietnam,^8^ evaluated the reasons for non-adherence. Respondents were TB contacts who did not adhere to their scheduled screening date. They had to select a response for each of the 16 statements. The 3rd section assessed TB knowledge. The 19 statements in this section were partly adapted and modified from an instrument used in a study conducted in South Africa.^7^ Respondents were both TB contacts who adhered and did not adhere to TB contact screening. Their TB knowledge was evaluated, specifically in terms of the causative factors, mode of transmission, aetiology, risk factors and at-risk groups, common symptoms and treatment.

**Validation and pilot study**

Approval was obtained from the authors of the 2 previous studies from which our questionnaire was adapted and modified.^7^'^8^ The questionnaire was translated into Malay by 2 bilingual experts. Back translation from the translated Malay version to the original English version was conducted by 2 native Malay speakers proficient in English.

The questionnaire items were reviewed for their content and face validities. Content validation was performed by an expert panel, comprising a physician from the respiratory unit of Sarawak General Hospital Kuching, a family medicine specialist from University Malaysia Sarawak and a medical officer from Tuberculosis Control Programme Kuching. Further content validation was conducted by a biostatistician from the Clinical Research Centre, Sarawak General Hospital. The questionnaires (in both English and Malay) were then provided to 15 TB contacts to ensure that the phrasing, terminology, layout and time taken to complete the questionnaires were clear, comprehensible and appropriate to our target population. All respondents understood the questionnaire items and provided appropriate feedback. Terminologies were understood. No reliability analysis was performed (e.g. test-retest validity) given the difficulty to get the same patient cohort to come back to recomplete the questionnaires, mostly stating reasons of time constraints.

### Data analysis

Data were analysed using IBM SPSS Statistics version 22 (IBM, Armonk, NY). Categorical data including the patient demographic characteristics, factors for non-adherence and knowledge responses were analysed using descriptive statistics (frequencies and percentages). The association between the patient demographic characteristics and adherence and between the knowledge responses and adherence was determined using the chi-square test. P<0.05 was considered statistically significant.

### Ethical considerations

For patient confidentiality, study numbers were used in the data collection forms instead of patient names. Hard copies of the collected data were stored manually in a locked cabinet, only accessible to the research team members.

## Results

Among 450 TB contacts eligible for our study, 383 participated, and 67 were excluded, yielding a response rate of 85%. The reasons for non-response were mainly refusal to be interviewed, time constraints and work commitments at the time of visit.

### Association between the demographic characteristics and follow-up adherence

Of the participants, 82.2% (n=315) were nonhousehold contacts, while 61.6% (n=234) were contacts from workplace colleagues. Most participants adhered to their 2nd TB contact screening (80.9%, n=310). Employment and ethnicity significantly influenced adherence. A significant majority of the participants were employed: 80.2% (n=247) in the group that adhered and 67.1% (n=49) in the group that did not (χ^2^=5.818, P=0.016). Malays comprised the significant majority: 51.9% (n=161) of those who adhered and 63.0% (n=46) of those who did not (χ^2^=11.601, P=0.009) (Table 1).

**Table 1:** Association between the demographic characteristics and follow-up adherence.

	n	Adherence n (%)	Non-adherence n (%)	χ^2^ (df)	P
**Age, year** <20 20-29 30-39 40-49 50-59 >60	25 120 97 73 46 22	23 100 72 62 35 18	2 20 25 11 11 4	6.729 (5)	0.242
**Sex** Male Female	169 214	138 (44.5) 172 (55.5)	31 (42.5) 42 (57.5)	0.101 (1)	0.751
**Marital status** Single Married Divorced/widowed	163 206 14	136 (43.9) 165 (53.2) 9 (2.9)	27 (37.0) 41 (56.2) 5 (6.8)	3.270 (2)	0.195
**Relationship with the index patient** Spouse Sibling Parent Other relative Colleague Others (not otherwise specified)	21 15 33 39 248 27	15 (4.8) 10 (3.2) 25 (8.1) 32 (10.3) 209 (67.4) 19 (6.1)	6 (8.2) 5 (6.8) 8 (11.0) 7 (9.6) 39 (53.4) 8 (11.0)	7.560 (5)	0.182
**Type of contact**					
Household	68	51 (16.5)	17 (23.3)	1.891 (1)	0.169
Non-household	315	**259 (83.5)**	**56 (76.7)**		
**Non-household contact**					
Workplace colleague Same school	234 35	199 25	35 10	6.735 (4)	0.151
Social^*^	45	36	9		
Institutional^**^	3	2	1		
Not applicable	63	47	16		
**Occupational status** Unemployed	85	61 (19.8)	24 (32.9)	5.818 (0.016)	**0.016**
Employed	296	**247 (80.2)**	**49 (67.1)**		
**Educational level**					
Primary school and below Secondary school	26 183	22 (7.2) 149 (48.9)	4 (5.5) 34 (46.6)	0.532 (2)	0.766
Tertiary education and above	169	134 (43.9)	35 (47.9)		
**Ethnicity** Malay	207	**161 (51.9)**	**46 (63.0)**		
Chinese	47	33 (10.6)	14 (19.2)	11.601 (3)	0.009
Bumiputera Sarawak	120	108 (34.8)	12 (16.4)		
Others^***^	9	8 (2.6)	1 (1.4)		
**Income**					
Below RM 1000 (very low)	75	60(19.4)	15 (20.5)		
RM 1001-3000 (low) RM 3001-5000 (middle)	193 50	164 (52.9) 41(13.2)	29 (39.7) 9 (12.3)	8.054 (4)	0.090
Above RM 5000 (high)	24	16 (5.2)	8 (11.0)		
No response	41	29 (9.4)	12 (16.4)		
**Smoking status** Current smoker Previous smoker	95 37	80 (25.9) 30 (9.7)	15 (20.5) 7 (9.6)	0.948 (2)	0.623
Never-/passive smoker	250	199 (64.4)	51 (69.9)		

* Social contacts: relatives of different households but who meet frequently

** Institutional contacts: nursing/care home or prison facilities

*** Other ethnicity: Indians, non-Malaysians and other races not otherwise specified

### Reasons for non-adherence

Of the 73 non-adherent TB contacts, 69 responded to the questionnaire section regarding the reasons for non-adherence. Among them, 52.1% (n=36) cited forgetting their scheduled appointment date as a factor for non-adherence. Approximately 39.1% (n=27) were aware of their risk for active TB. For the other reasons, most responded being unsure (Table 2).

**Table 2:** Reasons for non-adherence.

	Reasons for non-adherence (n=69)	Strongly agree n (%)	Agree n (%)	Not sure n (%)	Disagree n (%)	Strongly disagree n (%)
Q1	I forgot my scheduled appointment date	19 (27.5)	17 (24.6)	19 (27.5)	12 (17.4)	2 (2.9)
Q2	I found the clinic (PR1 unit) staff unhelpful	0	2 (2.9)	44 (63.8)	19 (27.5)	4 (5.8)
Q3	I did not clearly understand the follow-up instructions given	2 (2.9)	7 (10.1)	37 (53.6)	20 (29)	3 (4.3)
Q4	My initial screening was negative, so I did not see the need for further screening	4 (5.8)	19 (27.5)	24 (34.8)	9 (13)	13 (18.8)
Q5	I had a difficulty to get time off other duties	8 (11.6)	9 (13.0)	35 (50.7)	10 (14.5)	7(10.1)
Q6	I was not in town during the given date	1 (1.4)	7 (10.1)	37 (53.6)	18 (26.1)	6 (8.7)
Q7	I have relocated my place of stay/work	2 (2.9)	5 (7.2)	37 (53.6)	21 (30.4)	4 (5.8)
Q8	I was busy with my personal affairs	6 (8.7)	16 (23.2)	28 (40.6)	16 (23.2)	3 (4.3)
Q9	I am afraid of contracting active TB or of having positive findings	4 (5.8)	14 (20.3)	27 (39.1)	19 (27.5)	5 (7.2)
Q10	I did not have transportation.	4 (5.8)	3 (4.3)	37 (53.6)	22 (31.9)	3 (4.3)
Q11	I chose to follow up at a private hospital or clinic.	2 (2.9)	4 (5.8)	41 (59.4)	18 (26.1)	4 (5.8)
Q12	I am concerned about radiation from chest radiography (conducted every follow-up)	2 (2.9)	10 (14.5)	33 (47.8)	14 (20.3)	10 (14.5)
Q13	I feel that a total of four follow-up visits are unnecessary	2 (2.9)	15 (21.7)	31 (44.9)	13 (18.8)	8 (11.6)
Q14	I am not aware of my risk of developing active TB	2 (2.9)	14 (20.3)	26 (37.7)	15 (21.7)	12 (17.4)
Q15	An incorrect appointment date was given	1 (1.4)	0	38 (55.1)	24 (34.8)	6 (8.7)
Q16	No appointment date was given	4 (5.8)	5 (7.2)	35 (50.7)	21 (30.4)	4 (5.8)

### TB knowledge

Most participants (81.7%, n=313) agreed that TB is caused by a bacterial infection. Conversely, 26.2% (n=100) incorrectly believed that it is inherited. TB symptoms were recognised correctly by most participants: prolonged cough (89.3%, n=342), unintentional weight loss (87.7%, n=336) and haemoptysis (86.4%, n=331). The majority of the participants were aware that TB can be spread via the following modes of transmission: sneezing (88.3%, n=338); hugging and kissing (79.1%, n=302); and talking (67.1%, n=257). Nearly half (49.1%, n=187) did not recognise diabetes mellitus (DM) as a risk factor, and 49.5% (n=189) were unsure whether TB can be cured with proper treatment (Table 3).

**Table 3:** TB knowledge (N=383).

Rank	Statement no.	Knowledge-related statements	Correct response n (%)	Incorrect response n (%)	Not sure n (%)
1	S14	Symptom commonly associated with TB: prolonged cough for more than 2 weeks (true)	342 (89.3)	31 (8.1)	10 (2.6)
2	S5	TB can be transmitted by sneezing (true)	338 (88.3)	37 (9.7)	8 (2.1)
3	S15	Symptom commonly associated with TB: unintentional weight loss (true)	336 (87.7)	38 (9.9)	9 (2.3)
4	S16	Symptom commonly associated with TB: haemoptysis (true)	331 (86.4)	43 (11.2)	9 (2.3)
5	S1	TB is caused by a bacterial infection (true)	313 (81.7)	55 (14.4)	15 (3.9)
6	S10	TB can be transmitted by hugging or kissing (true)	302 (79.1)	52 (13.6)	28 (7.3)
7	S19	TB can cause death (true)	300 (78.3)	56 (14.6)	27 (7.0)
8	S6	TB can be transmitted by talking (true)	257 (67.1)	63 (16.4)	63 (16.4)
9	S4	Smoking increases my risk for TB (true)	254 (66.3)	77(20.1)	52 (13.6)
10	S11	The risk for TB is increased among children aged under 5 years (true)	245 (64.0)	108 (28.2)	30 (7.8)
11	S12	The risk for TB is increased among patients positive for HIV (true)	205 (53.5)	147 (38.4)	31 (8.1)
12	S18	Herbal or traditional medicine can cure TB (false)	187 (48.8)	85 (22.2)	111 (29.0)
13	S17	TB cannot be cured with proper treatment (false)	150 (39.3)	43 (11.3)	189 (49.5)
14	S13	The risk for TB is increased among patients with diabetes mellitus (true)	141 (36.8)	187(49.1)	53 (13.9)
15	S3	TB is inherited (false)	129 (33.8)	100 (26.2)	153 (40.1)
16	S9	TB can be transmitted by sharing towels, clothes, etc (false)	94 (24.6)	205 (53.7)	83 (21.7)
17	S7	TB can be transmitted by sharing utensils (false)	84 (22.0)	223 (58.5)	74 (19.4)
18	S8	TB can be transmitted by sleeping in the same bedroom (false)	82 (21.5)	240 (62.8)	60 (15.7)
19	S2	TB is caused by living in an unhygienic environment (false)	77(20.1)	222 (58.0)	84 (21.9)

The highest rank is determined by the highest percentage of correct responses.

### Association between TB knowledge and follow-up adherence

Both groups correctly described the aetiology of TB. Approximately 88.2% (n=262) and 71.8% (n=51) of the participants who adhered and did not, correctly answered that TB is caused by a bacterial infection, respectively. This association was statistically significant (χ^2^=12.101, P=0.001). Approximately 78.9% (n=195) of the participants who adhered and 51.9% (n=27) of those who did not, incorrectly answered that TB is caused by living in an unhygienic environment. This association was also highly significant (χ^2^=16.407, P<0.001) (Table 4).

**Table 4:** TB knowledge and follow-up adherence.

Variable	n	Adherence n (%)	Non-adherence n (%)	χ^2^(df)	P
TB is caused by a bacterial infection Correct response Incorrect response	313 55	262 (88.2) 35 (11.8)	51 (71.8) 20 (28.2)	12.101 (1)	**0.001**
TB is caused by living in an unhygienic environment Correct response Incorrect response	77 222	52 (21.1) 195 (78.9)	25 (48.1) 27 (51.9)	16.407 (1)	**0.001**
TB is inherited Correct response Incorrect response	129 100	100 (53.5) 87 (46.5)	29 (69.0) 13 (31.0)	3.381 (1)	0.066
Smoking increases my risk for TB Correct response Incorrect response	254 77	209 (78.6) 57(21.4)	45 (69.2) 20 (30.8)	2.553 (1)	0.110
TB can be transmitted by sneezing Correct response Incorrect response	338 37	276 (90.8) 28 (9.2)	62 (87.3) 9 (12.7)	0.777 (1)	0.378
TB can be transmitted by talking Correct response Incorrect response	257 63	207 (79.6) 53 (20.4)	50 (83.3) 10 (16.7)	0.426 (1)	0.514
TB can be transmitted by sharing utensils Correct response Incorrect response	84 223	66 (26.3) 185 (73.7)	18 (32.1) 38 (67.9)	0.788 (1)	0.375
TB can be transmitted by sleeping in the same bedroom Correct response Incorrect response	82 240	65 (25.2) 193 (74.8)	17 (26.6) 47 (73.4)	0.051 (1)	0.822
TB can be transmitted by sharing towels, clothes, etc Correct response Incorrect response	94 205	73 (30.5) 166 (69.5)	21 (35.0) 39 (65.0)	0.442 (1)	0.506
TB can be transmitted by hugging or kissing Correct response Incorrect response	302 52	248 (86.4) 39 (13.6)	54 (80.6) 13 (19.4)	1.465 (1)	0.226
The risk for TB is increased among children aged under 5 Correct response Incorrect response	245 108	201 (70.0) 86 (30.0)	44 (66.7) 22 (33.3)	0.287 (1)	0.592
The risk for TB is increased among patients positive for HIV. Correct response Incorrect response	205 147	167 (59.4) 114 (40.6)	38 (53.5) 33 (46.5)	0.814 (1)	0.367
The risk for TB is increased among patients with diabetes mellitus. Correct response Incorrect response	141 187	113(43.1) 149 (56.9)	28 (42.4) 38 (57.6)	0.011 (1)	0.918
Symptom commonly associated with TB: prolonged cough (*more than 2 weeks of duration*) Correct response Incorrect response	342 31	279 (92.7) 22 (7.3)	63 (87.5) 9 (12.5)	2.055 (1)	0.152
Symptom commonly associated with TB: unintentional weight loss Correct response Incorrect response	336 38	272 (89.8) 31 (10.2)	64 (90.1) 7 (9.9)	0.009 (1)	0.926
Symptom commonly associated with TB: haemoptysis Correct response Incorrect response	331 43	270 (88.8) 34 (11.2)	61 (87.1) 9 (12.9)	0.156 (1)	0.692
TB cannot be cured with proper treatment. Correct response Incorrect response	150 43	122 (78.2) 34 (21.8)	28 (75.7) 9 (24.3)	0.111 (1)	0.740
Herbal or traditional medicine can cure TB. Correct response Incorrect response	187 85	148 (67.9) 70 (32.1)	39 (72.2) 15 (27.8)	0.378 (1)	0.539
TB can cause death. Correct response Incorrect response	300 56	246 (84.8) 44 (15.2)	54 (81.8) 12 (18.2)	0.367 (1)	0.544

TB, tuberculosis

## Discussion

### Demographic characteristics influencing adherence

Our study found that 19.1% of the TB contacts did not adhere to their 2nd TB contact screening appointment date. A similar rate of non-adherence was observed in a study in Vietnam, where 13% (n=109) of TB contacts did not adhere to follow-up. The study reported similar participant demographics.^8^ A study conducted in South Africa^7^ found that male sex and advancing age were significantly associated with nonadherence. However, that study focused only on household contacts who did not adhere to follow-up screening and reported a non-adherence rate of 52.9%. Similarly, in Ethiopia,^9^ the non-adherence rate was reported as 66.7%, which is higher than that in our study. However, the contact screening adherence in that study was significantly associated with religion, family income, relationship with contact and family support.

### Factors associated with non-adherence

This study identified that the reason for non-adherence was mainly forgetting their appointment date (52.1%, n=36) given the long 8-month intervals of follow-up from the initial TB contact screening.

Similarly, in Vietnam,^8^ 70% (n=73) of participants who did not adhere to follow-up explained that they initially forgot their scheduled appointment. Meanwhile, in South Africa,^7^ 44.4% (n=20) of contacts reported difficulties to get time off other duties. This factor was not a selected response for our study participants.

### TB knowledge and non-adherence

For the aetiology of TB, most TB contacts from both groups correctly responded that TB is caused by a bacterial infection. A correct response rate of 88.2% (n=262) and 71.8% (n=51) was obtained from those who adhered and who did not, respectively. This finding could be due to the participants’ reasonably good educational level. Our findings are in line with those in a Vietnam study^8^ that found 68% (n=126) and 55% (n=56) of those who adhered and did not adhere to follow-up, respectively, correctly describing TB aetiology. In South Africa,^7^ 87.3% (n=124) of TB contacts also correctly described TB aetiology. Studies conducted on the knowledge and awareness of TB among students in Malawi, Ethiopia and India reported 90%, 81.7% and 81% of their participants being aware that bacteria cause TB, respectively.^10-12^

Most participants were aware of TB symptoms regardless of their adherence to follow-up. Specifically, both groups demonstrated high levels of recognition for prolonged cough lasting more than 2 weeks, unintentional weight loss and haemoptysis. Our findings are similar to those of a study performed in Sabah, East Malaysia.^5^

For the transmission mode, 78.9% (n=195) and 51.9% (n=27) of the TB contacts who adhered and did not adhere, respectively, incorrectly believed that TB is caused by living in an unhygienic environment. This finding could be due to the perception that an unhygienic environment is associated with bacterial growth, indicating a lack of public awareness on the transmission mode of TB, which is airborne. A study in Vietnam^8^ showed similar responses in 75% (n=139) and 83% (n=85) of contacts who adhered and did not adhere to follow-up, respectively. Indeed, overcrowding, unhealthy lifestyle, residence in close quarters with poor ventilation, poor nutrition and neglected hygiene among patients with TB are the main contributors for ongoing TB transmission, although an unhygienic environment alone does not directly contribute to it.

The participants’ understanding of what groups are at risk for TB varied. Notably, nearly half of our participants (49.1%, n=187) failed to recognise DM, a prevalent non-communicable disease in our population, as an important risk factor. This finding is concerning, as there is a two-to-four-fold higher risk of active TB in individuals with DM, and up to 30% of individuals with TB are likely to have DM.^13^

As for TB treatment, 51.1% (n=196) of the participants responded incorrectly or were unsure whether herbal or traditional medication can cure TB. This finding suggests that the Malay community still has local traditional influences as part of the deeply rooted practice of traditional medicine among Malay culture.^14,15^ Similarly, in Sabah, East Malaysia, 38% of TB contacts believed in the effectiveness of traditional medications for TB treatment, while 56% were uncertain.^5^ In South Africa, 65.5% of TB contacts incorrectly believed in the curative properties of herbal medicine for TB, likely influenced by the high regard and strong beliefs in traditional healers within certain communities.^7^ Conversely, a study in Vietnam reported that only 11% and 6% of individuals who adhered and did not adhere to follow-up, respectively, believed that traditional medications can cure TB.^8^

If TB contacts are unsure about the benefits of conventional treatment, they may be less motivated to adhere to contact screening activities, assuming that traditional healing methods might suffice or be equally effective. Consequently, this can lead to delays in diagnosis, increased transmission rates and challenges in controlling TB within communities.

### Implications for future research

As most participants cited forgetting their scheduled appointment as a factor for non-adherence, this potential barrier to completing their follow-up screening needs to be addressed. We suggest establishing innovative strategies such as scheduled earlier phone calls or written follow-up reminders several weeks before patients’ subsequent TB contact screening appointment dates. Overall, there was acceptable knowledge of common TB symptoms but mixed response on how TB can be transmitted. The fact that DM was not recognised as a risk factor by almost half of the participants shows that more attention should be given to these patients to inform them of their risk. Future public health measures should be undertaken to ensure that the public is given clear and accurate information, especially on TB’s airborne transmission, and identify areas for improvement in the information delivery at all levels. It is also important to emphasise that TB can potentially lead to death but is curable with proper treatment, not through unproven herbal or traditional treatments.

As this study mainly focused on patient-related factors influencing follow-up adherence, future studies are suggested to analyse healthcare worker and service-related factors, efficacy of our current TB contact defaulter system, including ways to improve engagement with our local Health Inspectors. Psychosocial factors, such as the potential stigma of being a TB contact, should also be studied. A qualitative study design should be adopted to further explore the exact reasons for nonadherence and the factors affecting TB knowledge. Future studies should also evaluate the incidence of TB among contacts during the first year following exposure. This is relevant, as the incidence of new cases is the highest in the 1st year after exposure to a patient with TB.^7^

### Strengths and limitations

To our knowledge, this study is the first of its kind to be conducted in Sarawak, East Malaysia. The results provide general insights into the barriers to adherence and the TB knowledge of the local patient population. However, the study was conducted in a single setting, and the majority of the participants were Malays (as the local residents the clinic serves are mostly Malays). Owing to ethical and practical reasons, we were unable to evaluate the response of eligible TB contacts who were unwilling to participate and defaulters who did not turn up despite the clinic staff’s multiple attempts to contact them through phone calls.

Another potential limitation is that most participants provided a neutral response for the reasons for non-adherence and TB knowledge, perhaps due to finding some of the questions too sensitive to be answered. There may also be social desirability bias among the participants, towards which they assumed a good response. Another major limitation is the sample size calculation based on a non-adherence rate of 68%. Our study noted a non-adherence rate of 19.1%, possibly affecting the validity of the findings.

## Conclusion

Our study findings show that there are improvements to be made in our reminder notifications and ‘call back’ system for TB contacts to improve adherence, especially when there is such long screening interval. There is also a need to disseminate accurate information about TB among the public, focusing on the modes of disease transmission. Future public health interventions (alongside health inspection) could include innovative reminder systems involving digital technology.

## Data sharing statement

Data are available upon request to the principal investigator.

**How does this paper make a difference in general practice?**
This study identified forgetfulness, knowledge gaps, and lack of awareness about comorbidities as factors hindering TB contact follow-up adherence. It highlighted the need for improved appointment reminder notifications, patient education on TB transmission and treatment, awareness of DM as a risk factor, and need to improve engagement with Public Health services.
